# Use of Antipsychotics in Emotionally Unstable Personality Disorder

**DOI:** 10.1192/bjo.2024.595

**Published:** 2024-08-01

**Authors:** Deepak Kuriakose

**Affiliations:** Wrexham Maelor Hospital, Wrexham, United Kingdom

## Abstract

**Aims:**

Emotionally unstable personality disorder (EUPD) is characterized by affective instability, unstable interpersonal relationships, poor self-image and marked impulsivity. Patients may present with a variety of symptoms including impulsivity, suicidal behavior, affective instability and intense anger. This makes the treatment very patient specific.

Treatment guidelines support the use of Dialectical Behavior Therapy (DBT) as the first line treatment of EUPD. Currently, no medications are indicated for the treatment of EUPD which leads to off label use of medicines by clinicians.

More than 90% of individuals receive a variety of pharmacologic treatment with psychotropic medications, especially second-generation antipsychotic drugs for the treatment of cognitive perceptual symptoms and impulse control behavior. Additional psychotropics are usually added leading to psychotropic polypharmacy which should be avoided.

Aim of this study is to assess the frequency of prescription of antipsychotic medications in patients with a primary diagnosis of emotionally unstable personality disorder.

**Methods:**

Protocol was registered with the Audit and Quality Improvement project team of the NHS trust and the audit registration certificate was obtained.

Case records of 42 patients with EUPD who attended psychiatric outpatient department from June to August 2023 were collected and screened. A retrospective study was carried out.

Inclusion criteria

Patients above 18 years of age, with a primary diagnosis of emotionally unstable personality disorder.

Exclusion criteria

Patients with comorbid diseases like Attention Deficit Hyperactivity Disorder, Bipolar Affective Disorder and Psychosis where use of antipsychotics is warranted.

All other personality disorders.

After screening 42 case records, 20 cases of EUPD which fulfilled the inclusion and exclusion criteria were found and analyzed. Descriptive statistics were used.

**Results:**

Retrospective data of 20 patients with a primary diagnosis of EUPD were analyzed which included 18 females and 2 males. The mean age of the participants was 27.1.

70% (14) of the patients diagnosed with EUPD were treated with antipsychotics. 20% (4) patients received antidepressants. 10% (2) of the patients received only DBT.

Quetiapine was the most commonly used antipsychotic – 43% (6) followed by Olanzapine – 22% (3), Risperidone – 21% (3) and Zuclopenthixol long-acting injection – 14% (2).

**Conclusion:**

Dialectical behavior therapy is the first line treatment of EUPD. National Institute for Health and Care Excellence (NICE) guidelines do not recommend the use of antipsychotics in the treatment of EUPD. Contrary to the guidelines, antipsychotics are prescribed long term for patients with EUPD who are without any comorbid conditions. This audit has found that 70% of patients with a primary diagnosis of EUPD are being prescribed antipsychotic medication. This needs to be kept in check so that polypharmacy can be avoided.

